# Mutant HRAS as novel target for MEK and mTOR inhibitors

**DOI:** 10.18632/oncotarget.5619

**Published:** 2015-11-03

**Authors:** Michael K. Kiessling, Alessandra Curioni-Fontecedro, Panagiotis Samaras, Kirstin Atrott, Jesus Cosin-Roger, Silvia Lang, Michael Scharl, Gerhard Rogler

**Affiliations:** ^1^ Division of Gastroenterology and Hepatology, University Hospital Zurich, and University of Zurich, Zurich, Switzerland; ^2^ Division of Oncology, University Hospital Zurich, and University of Zurich, Zurich, Switzerland; ^3^ Department of Pharmacology and CIBERehd, Faculty of Medicine, University of Valencia, Valencia, Spain

**Keywords:** HRAS mutations, MEK inhibitor, mTOR inhibitor, lung cancer, bladder cancer

## Abstract

HRAS is a frequently mutated oncogene in cancer. However, mutant HRAS as drug target has not been investigated so far. Here, we show that mutant HRAS hyperactivates the RAS and the mTOR pathway in various cancer cell lines including lung, bladder and esophageal cancer. HRAS mutation sensitized toward growth inhibition by the MEK inhibitors AZD6244, MEK162 and PD0325901. Further, we found that MEK inhibitors induce apoptosis in mutant HRAS cell lines but not in cell lines lacking RAS mutations. In addition, knockdown of HRAS by siRNA blocked cell growth in mutant HRAS cell lines. Inhibition of the PI3K pathway alone or in combination with MEK inhibitors did not alter signaling nor had an impact on viability. However, inhibition of mTOR or combined inhibition of MEK and mTOR reduced cell growth in a synergistic manner. Finally, Ba/F3 cells transformed with mutant HRAS isoforms Q61L, Q61R and G12V demonstrated equal sensitivity towards MEK and mTOR inhibition. Our results show that HRAS mutations in cancer activate the RAS and mTOR pathways which might serve as a therapeutic option for patients with HRAS mutant tumors.

## INTRODUCTION

Harvey-RAS (HRAS) belongs to the RAS family of small GTPases which activate the RAS–RAF–MEK–ERK pathway. Mutations in RAS family members lead to hyperactivity of the RAS signaling pathway. HRAS is a frequently mutated oncogene especially in head and neck cancer (3.9%), bladder cancer (5.1%), vulvar squamous cell carcinoma (9.3%), cutaneous squamous cell carcinoma and lung cancer (3.8%) [1–3]. This adds up to a significant number of patients eligible for putative targeted therapies. Frequency of HRAS mutations varies. Histological subtypes could play a role as a report described a high frequency of HRAS mutations in inverted urothelial papilloma (IUP) - an uncommon neoplasm of the urinary bladder with distinct morphologic features [4]. In addition, HRAS mutations seem to be more frequent in squamous cell cancer of the lung (2.8%) than in adenocarcinoma of the lung (1%) [2, 5]. Clinical characteristics and behavior of HRAS mutant cancer patients have been described scarcely. One recent report described an adenocarcinoma of the lung with HRAS Q61L mutation suffering from rapid progression and deterioration suggesting that HRAS mutations in NSCLC tumors might be aggressive and associated with poor overall prognosis, similar to KRAS mutant NSCL [6]. Interestingly, one phase I trial for the novel Mitogen-activated protein kinase kinase (MEK) inhibitor RO5126766 reported a tumor patient with HRAS mutation that showed 20% tumor shrinkage due to MEK inhibitor treatment [7]. This is the first hint that HRAS mutant cancer patients might benefit clinically from MEK inhibitor treatment. However, whether HRAS mutations generally sensitize towards treatment with MEK inhibitors has not been investigated yet. Further, little is known about signaling of oncogenic HRAS and putative druggability. Typical hotspots for HRAS mutations are found at codon 12, 13 and 61, resulting in G12C/S, G13R/V and Q61R/L mutations [8]. These positions for mutations of HRAS are at the very same sides as mutations found for NRAS [9]. As of today, mutant NRAS has been far better investigated, mainly in melanoma but also in other cancers such as lung cancer and T-cell lymphoma. NRAS mutations are mostly found at codon 61 and to a fewer extend at codons 12 and 13. NRAS mutations occur at about 15% to 25% in melanoma patients [10, 11] and are known to activate the RAS–RAF–MEK–ERK pathway. Approaches targeting oncogenic NRAS directly including farnesylation inhibitors have failed. But inhibiting downstream MEK kinase by MEK kinase inhibitors was proven to be successful in melanoma, lung cancer and T-cell lymphoma cell lines [12–14]. MEK inhibitors blocked cell growth at clinical relevant concentrations and even induced apoptosis [12–14]. Mutant NRAS was also shown to activate the PI3K/mechanistic target of rapamycin (mTOR)-signaling cascade and combined inhibition of MEK and PI3K was synergistic in certain NRAS mutant cell lines of melanoma, lung cancer and neuroblastoma [14, 15]. More important, the concept of targeted treatment of NRAS mutant melanoma could be demonstrated within clinical trials. In a phase II trial 30 patients with NRAS mutant melanoma were treated with the MEK inhibitor MEK162. 20% of treated patients showed a partial response and 43% stable disease [16]. These promising results will be further studied in a phase III clinical trial [17].

In the present study, we investigated HRAS downstream signaling in five different cancer cell lines including lung and bladder cancer and putative drugs for targeted therapy. We observed that lung cancer cell lines harboring HRAS mutations showed significant higher sensitivity to MEK inhibitors than HRAS wild-type cell lines. Further, the effect of MEK inhibitors was enhanced synergistically by the addition of mTOR but not PI3K inhibitors. Inhibition of mTOR alone is sufficient to block cell growth in HRAS mutant but not wild-type cell lines. To our knowledge, we describe for the first time that HRAS mutations can be targeted by MEK and mTOR inhibitors.

## RESULTS

### MEK inhibitors block cell growth in HRAS mutant cancer cell lines

**Figure 1:**
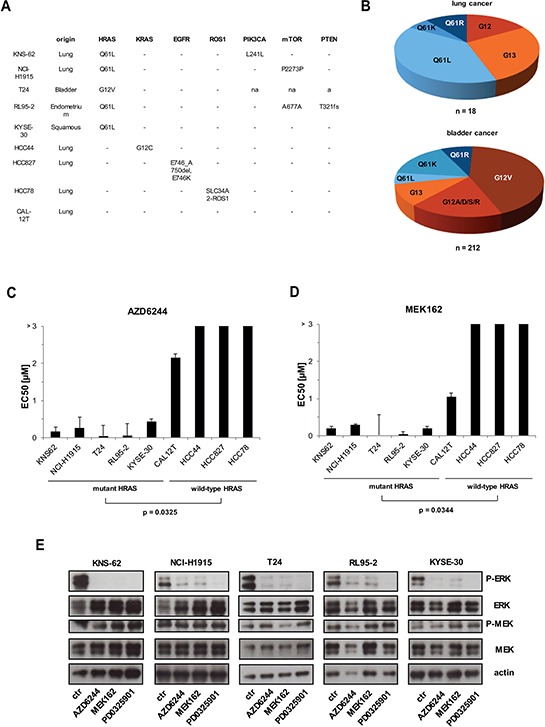
MEK inhibition blocks cell growth in HRAS mutant cells **A.** Presentation of HRAS mutant and HRAS wild-type cell lines. Mutations were detected by PCR of cDNA covering the open reading frame of HRAS and confirmed mutation status from COSMIC data base. PCR products were purified and sent for sequencing. **B.** Distribution of HRAS mutations from tumor tissue of lung (upper panel) and bladder (lower panel) available from COSMIC data base. **C, D.** Mutant HRAS und wild-type HRAS cell lines were treated with 6 increasing concentrations of AZD6244 and MEK162 for 96 hours. Then, cell growth was measured by Cell Titer Glo according to the manufacturer's instructions. EC50 values were calculated with GraphPad Prism and depicted at bars. Statistical significance between mutant and wild-type cell lines was calculated with student's *t*-test. **E.** All cell lines were kept under equal conditions, then treated with 500nM of AZD6244, MEK162 and PD0325901 for 1 hour and next lysed and subjected to Western blot. Phosphorylation levels of ERK and MEK were detected by specific anti-phospho antibodies. Loading was verified by specific antibodies to total ERK, MEK and anti–actin.

In order to study HRAS mutations we collected HRAS mutant cancer cell lines including KNS-62, NCI-H1915, T24, RL95–2 and KYSE-30 (Figure 1A). Sequencing data for primary tumor tissue available at COSMIC data base revealed that the HRAS Q61L mutation is the most common in lung cancer of adenocarcinoma and squamous cell cancer (Figure 1B). This is reflected by the two lung cancer cell lines KNS-62 (squamous) and NCI-H1915 (adenocarcinoma) which both harbor a Q61L mutation. The HRAS G12V mutation is predominant in bladder cancer which was detected in the T24 bladder cancer cell line (Figure 1B) [19]. HCC827 is a cell line with described EGFR mutation and HCC78 was found to harbor a ROS1 translocation [20, 21]. RAS kinases are well known to activate the RAS–RAF–MEK–ERK cascade and several MEK inhibitors are under clinical development including AZD6244 (Selumetinib) and MEK162 (Binimetinib) [16, 22]. This prompted us to ask whether MEK inhibitors could be of relevance for treatment of HRAS mutated patients. We detected that HRAS mutant cell lines KNS-62, NCI-H1915, T24, RL95–2 and KYSE-30 had significantly lower EC50 values for MEK inhibition than HRAS wild-type cells such as CAL12T, HCC44, HCC827 and HCC78 (Figure 1C,1D, Supplementary Figure S1A). EC50 value for HRAS wild type cell lines CAL12T was 2.15 μM and for HCC44, HCC827 or HCC78 EC50 was above 3 μM (Figure 1C). In contrast, HRAS mutant cell lines had EC50 values for AZD6244 ranging from 4–5nM (T24 and RL95–2) over 166–258nM (KNS-62, NCI-H1915) to 437nM (KYSE-30) (Figure 1C). EC50 values for MEK162 were found to be in a similar range (Figure 1D). These are clinical relevant plasma concentrations since maximal plasma concentrations of AZD6244 and MEK162 are at 1.75 μM and 1.13 μM, respectively [23, 24]. Next, we wanted to investigate efficacy of MEK inhibitors under serum-reduced and hypoxic conditions. Though cell growth was slightly impaired under serum-reduced conditions MEK inhibitors efficiently blocked cell growth in HRAS mutant cell lines (Supplementary Figure S1B). Under hypoxic conditions with 0.2% oxygen cell growth of controls was strongly impaired (data not shown) and, thus, MEK inhibitors were slightly less efficient than under standard cell culture conditions (Supplementary Figure S1B). Further, we checked whether inhibition of MEK blocks signaling of the RAS–RAF–MEK–ERK pathway. AZD6244, MEK162 and PD0325901 blocked basal ERK phosphorylation in all HRAS mutant cell lines (Figure 1E). ERK phosphorylation was not blocked in HRAS wild-type cell lines (Supplementary Figure S1C). Taken together this data shows, that HRAS mutation results in hyperactivation of the RAS pathway in cancer cell lines from various tissues and that this activation sensitizes towards treatment with MEK inhibitors.

### Mutant HRAS is critical for survival of mutant HRAS cancer cell lines

**Figure 2:**
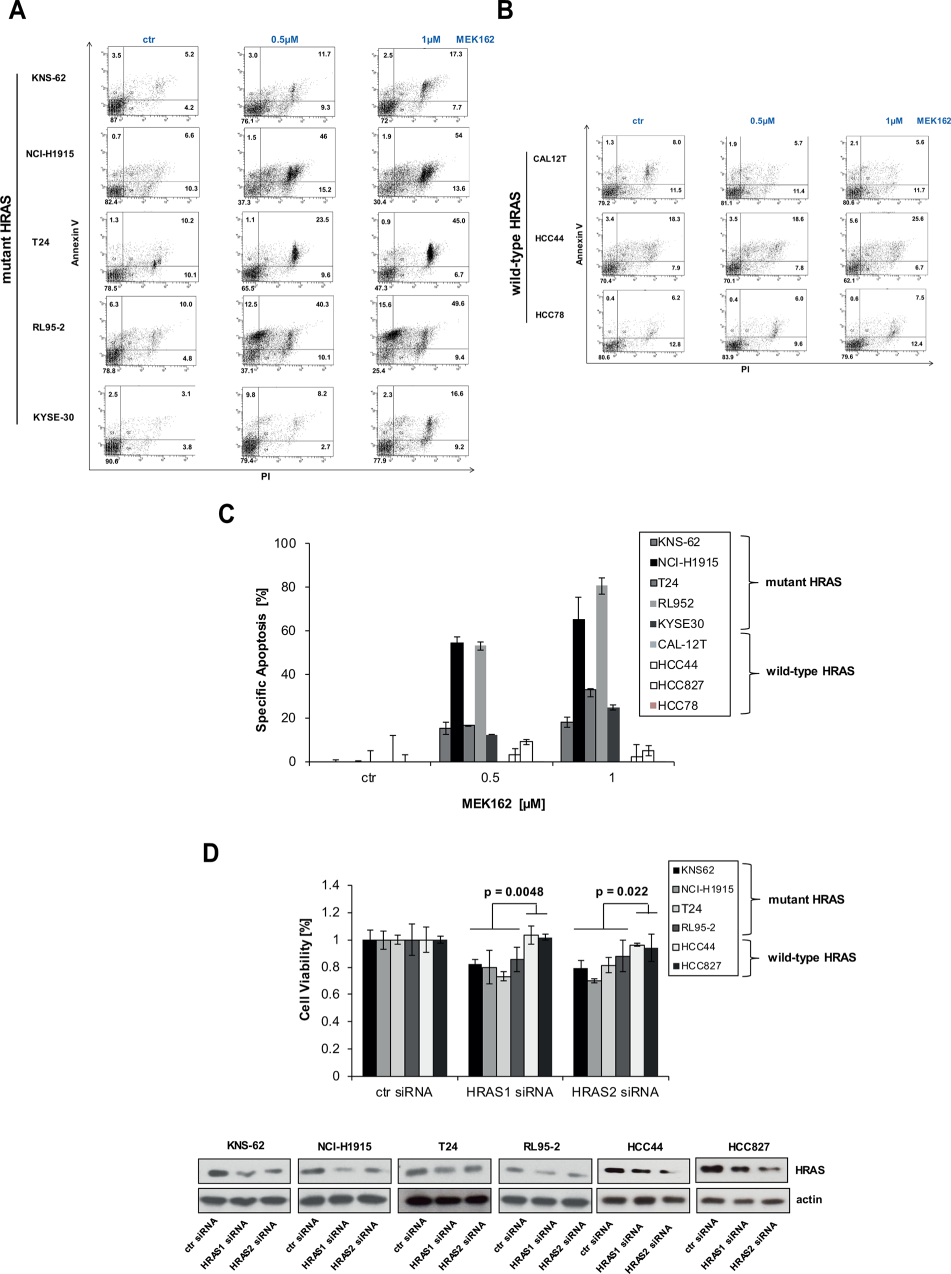
MEK inhibitors induce apoptosis in HRAS mutant cell lines **A.** HRAS mutant cell lines were incubated with indicated concentrations of MEK inhibitors MEK162 for 72 hours. Then, apoptosis was determined by Annexin V and PI staining. **B.** Same as A. but HRAS wild-type cell lines were used instead. **C.** Quantification of apoptosis by AnnexinV / PI assay. Specific apoptosis was calculated for all cell lines as described in M&M section. **D.** Accell siRNA against HRAS (siHRAS1 and siHRAS2) were transfected in HRAS mutant and HRAS wild-type cell lines according to manufacturer's instructions. 96 hours after transfection, cells were lysed and lysates subjected to Western blot. Efficiency of knockdown was measured by anti-HRAS antibodies. Equal loading was controlled by antiactin antibodies. Cell growth was also assessed after 96 h by Cell titer Glo. Statistical significance between mutant and wild-type cell lines was calculated with student's *t*-test.

Since MEK inhibitors block cell growth we wanted to analyze whether induction of apoptosis occurs in HRAS mutant cancer cell lines. We incubated cell lines with MEK inhibitors and measured apoptosis 72 hours later. Indeed, MEK inhibitor MEK162 induced apoptosis in HRAS mutant cell lines KNS-62, NCI-H1915, T24, RL95–2 and KYSE-30 but not in HRAS wild-type cell lines (Figure 2A, 2B). Apoptosis measured ranged between 18% and 60% for HRAS mutant cell lines (Figure 2C). To further study the role of HRAS, we performed a specific HRAS knockdown by 2 different siRNAs. HRAS siRNA reduced protein expression of HRAS which subsequently resulted in significantly reduced cell growth of HRAS mutant cell lines compared to wild-type cell lines (Figure 2D). These results show that HRAS mutant cell lines are addicted to continuous HRAS activity which is required for survival.

### Combined inhibition of MEK and mTOR synergistically reduces cell growth in HRAS mutant tumor cells

**Figure 3:**
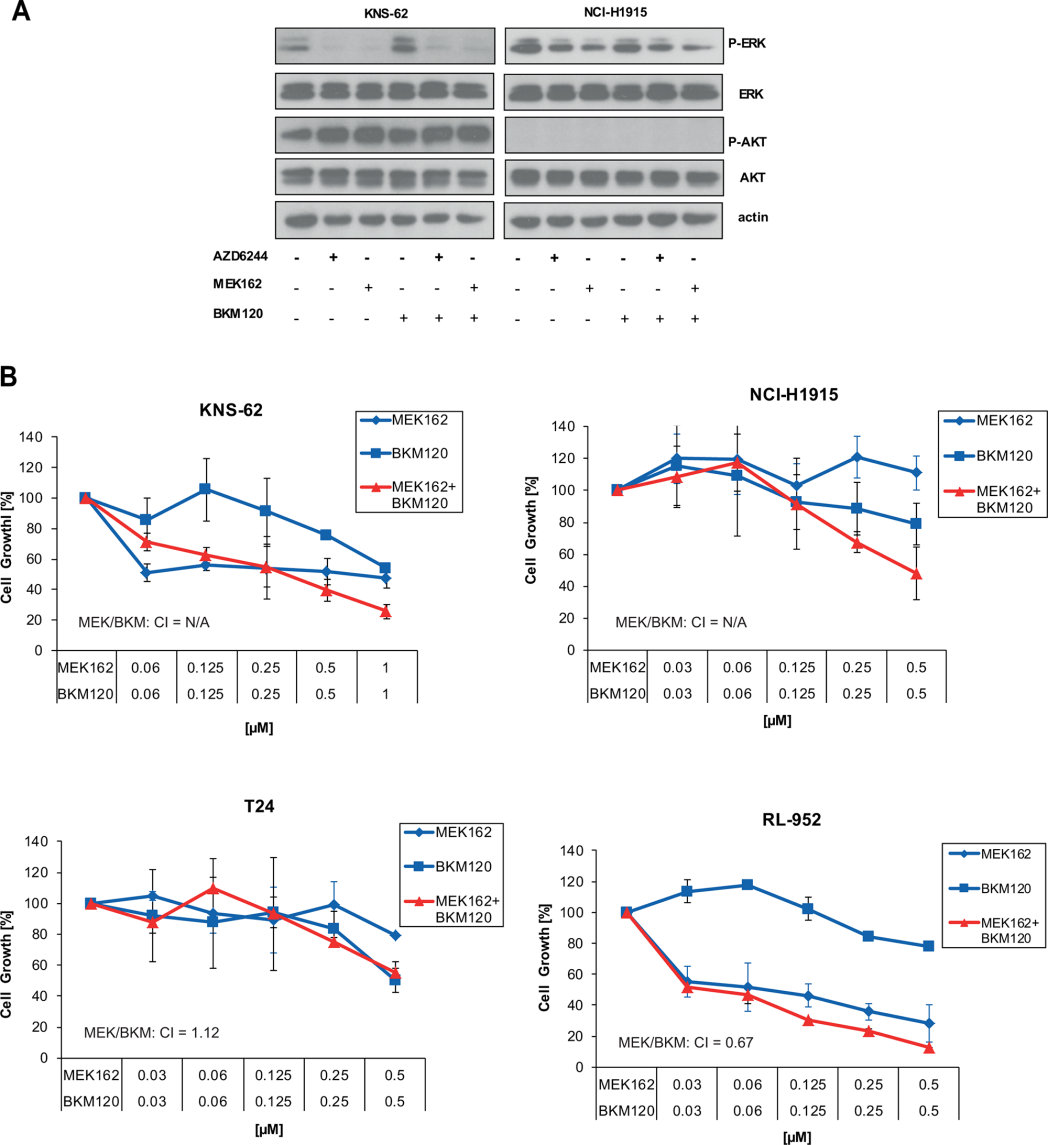
Inhibition of PI3K pathway does not influence signaling or cell growth **A.** KNS-62 and NCI-H1915 cells were treated with 500nM of AZD6244, MEK162 and BKM120 or combinations thereof as indicated for 1 h. Then, cells were lysed and analysed by Western blot. **B.** KNS-62, NCI-H1915, T24 and RL95–2 cells were left untreated or treated with indicated concentrations of MEK162 and BKM120 for 96 hours. Next, cell growth was measured by Cell Titer Glo according to the manufacturer's instructions. CIs are indicated.

Previously, it was demonstrated that mutant NRAS melanoma cells are sensitive to the combination of MEK and PI3K inhibitors [14]. We found that the NCI-H1915 cell line had no detectable phosphorylation levels of AKT Ser 473 despite strong expression of basal AKT (Figure 3A). Further, pan-PI3K inhibitor BKM120 did not alter phosphorylation of AKT Ser 473 in KNS-62 cell line (Figure 3A). These findings are in line with a lack of growth inhibition for single pan-PI3K inhibition or combined treatment with MEK162 in HRAS mutant cell lines (Figure 3B). These results were further supported by studies with a second PI3K kinase inhibitor BYL719 which had no effect on cell growth (data not shown). We conclude that inhibition of the PI3K pathway is not relevant for survival and cell growth of HRAS mutant cancer cells.

**Figure 4:**
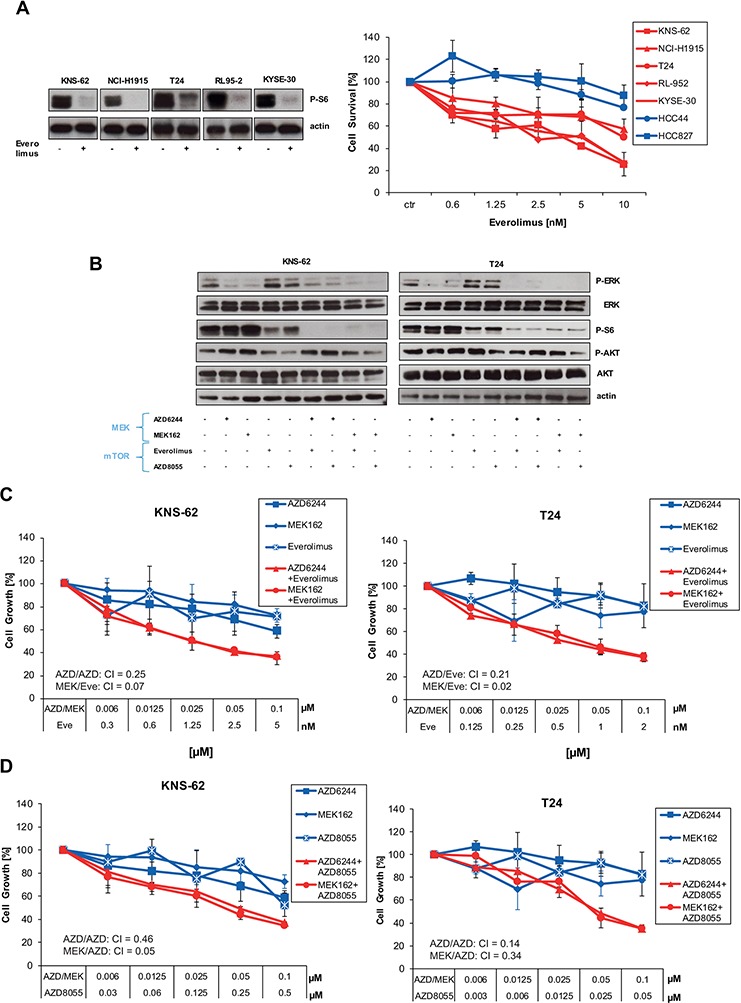
Inhibition of the mTOR pathway blocks cell growth in HRAS mutant cell lines **A.** HRAS mutant and wild-type cells were left untreated or treated with indicated concentrations of Everolimus for 96 hours. Next, cell growth was measured by Cell Titer Glo according to the manufacturer's instructions (left panel). In addition, all HRAS mutant cell lines were treated with 10nM Everolimus for 1 hour. Then, cells were lysed and analysed by Western blot applying phospho-S6 antibody and anti-actin antibodies for loading control (right panel). **B.** KNS-62 and T24 cells were treated with 250nM AZD6244, 250nM MEK162, 5nM of Everolimus, 250nM AKT8055 or combinations thereof as indicated for 1 hour. Then, cells were lysed and analysed by Western blot. CIs are indicated. **C.** KNS-62 and T24 cells were left untreated or treated with indicated concentrations of Everolimus and AZD6244/MEK162 for 96 hours. Then, cell growth was measured by Cell Titer Glo according to the manufacturer's instructions. CIs are indicated. **D.** Same as in C., but the mTOR inhibitor AZD8055 was used instead of Everolimus. CIs are indicated.

Enhanced apoptosis and tumor growth suppression by combination of MEK and mTOR inhibitors was previously observed in HRAS wild-type lung cancer cell lines [25]. Therefore, we asked the question whether inhibition of the down-stream mTOR complex could be an effective target for mutant HRAS. The clinically available mTOR inhibitor Everolimus blocked activity of S6, a kinase downstream of mTOR, in HRAS mutant cell lines (Figure 4A). This inhibition of the mTOR pathway translated in blockage of cell growth preferentially in HRAS mutant cancer cell lines (Figure 4A). Encouraged by these results, we aimed to investigate the combination of mTOR inhibitor Everolimus and MEK inhibitors. If used together, combination of Everolimus and AZD6244/MEK162 caused a stronger inhibition of S6 kinase than single use of Everolimus on Western blot (Figure 4B; Supplementary Figure S2A). The combination of Everolimus and AZD6244 / MEK162 also translated in a stronger blockade of cell growth in HRAS mutant cells than single use (Figure 4C; Supplementary Figure S2B). Of note, concentrations used for combined inhibition are at very low nanomolar concentrations for both Everolimus and AZD6244 or MEK162 (Figure 4C; Supplementary Figure S2B). More important, the combination of Everolimus and MEK inhibitors was found to be synergistic according to method of Chou-Talalay [18]. Synergism is defined by a combination index (CI) inferior 1 [18]. MEK162 showed stronger synergism with Everolimus than AZD6244 (Figure 4C). To further support the synergistic action of mTOR and MEK inhibitors in HRAS mutant cells we used the novel mTOR1/2 inhibitor AKT8055 which entered early clinical trials [26]. AKT8055 blocked downstream phosphorylation of S6 kinase alone (Figure 4B). Yet, phosphorylation of S6 kinase was further blocked if AKT8055 was used in combination with MEK inhibitors (Figure 4B). Combination of AKT8055 and MEK inhibitors also blocked cell growth synergistically in mutant HRAS cell lines according to Chou-Talalay (Figure 4D; Supplementary Figure S2C). To summarize, these data show that combination of mTOR and MEK inhibitors synergistically inhibit downstream signaling and cell growth of HRAS mutant cell lines.

### Mutant HRAS-driven transformation of Ba/F3 cells is sensitive to MEK inhibitors

**Figure 5:**
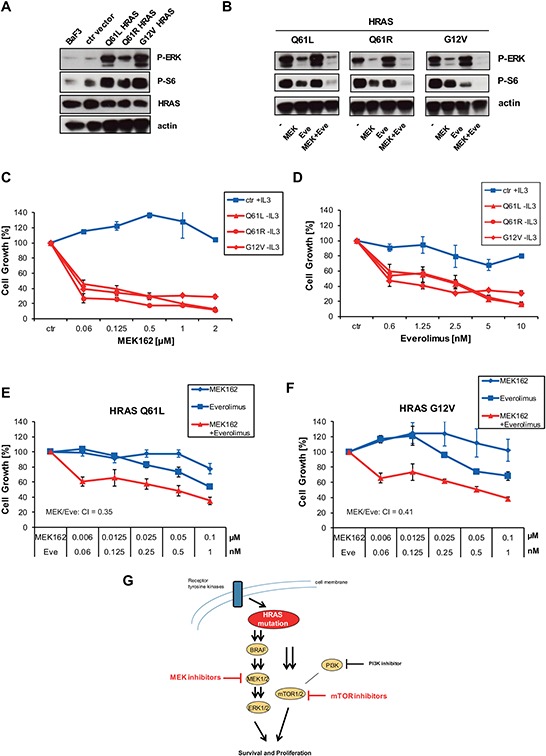
HRAS mutants transform Ba/F3 cells which become sensitive to MEK and mTOR inhibition **A.** Ba/F3 cells or Ba/F3 cells transduced with empty vector were kept under 0,1 ng/ml IL-3. Ba/F3 cells transduced with HRAS Q61L, Q61R or G12V grew independent of IL-3 and were kept under puromycin selection with 1ug/ml. Cells were lysed and analysed by Western blot with indicated antibodies. **B.** Ba/F3 clones were kept under same conditions as described in A. Cells were treated with 250nM AZD6244, 5nM of Everolimus or combinations thereof as indicated for 1 hour. Then, cells were lysed and analyzed by Western blot. **C.** Ba/F3 clones were kept under same conditions as described in A. Ba/F3 cells transduced with empty vector or HRAS Q61L, Q61R or G12V were treated with increasing concentrations of MEK162. Then, cell growth was measured by Cell Titer Glo after 96 h. **D.** Same as in C., but Everolimus was used instead. **E, F.** HRAS Q61L or G12V expressing clones were left untreated or treated with indicated concentrations of Everolimus and MEK162 for 96 hours. Then, cell growth was measured by Cell Titer Glo. CIs are indicated. **G.** Schematic representation of mutant HRAS signaling pathways. Mutant HRAS can be blocked by inhibition of downstream MEK and mTOR but not by PI3K.

We wanted to study the functional consequences of the different HRAS mutations including Q61L, Q61R and G12V in more detail by testing their abilities to transform interleukin-3 (IL-3)-dependent murine lymphoid Ba/F3 cells to cytokine-independent growth. Removal of IL-3 lead to a clear difference in cell proliferation. Ba/F3 cells expressing ectopic HRAS Q61L, Q61R or G12V mutations showed strong cell growth whereas Ba/F3 cells with wild-type HRAS or empty vector stopped cell growth (Supplementary Figure S3A). Ba/F3 cells expressing mutant versions of HRAS exhibited constitutive phosphorylation of ERK and S6 which was blocked by MEK162 or Everolimus, respectively (Figure 5A, 5B). All mutant HRAS forms including Q61L, Q61R and G12V sensitized equally towards MEK inhibitor or mTOR inhibitor treatment compared to control vector cell line kept under IL-3 (Figure 5C, 5D). The calculated EC50 values of transformed Ba/F3 cells were at low nanomolar range as observed for mutant cell lines. Since combined treatment of MEK and mTOR inhibitor resulted in stronger suppression of S6 phosphorylation than treatment with single inhibitors (Figure 5B), we wanted to check combined treatment on cell growth. In both HRAS mutants, Q61L and G12V, combination of MEK and mTOR inhibition caused a synergistic blockade of cell growth (Figure 5E, 5F). To conclude, these data clearly show that the different HRAS mutations at Q61 and G12 confer equal sensitivity towards inhibition of downstream MEK and mTOR pathway (Figure 5G).

### Combined inhibition of MEK and mTOR synergistically blocks tumor growth *in-vivo*

**Figure 6:**
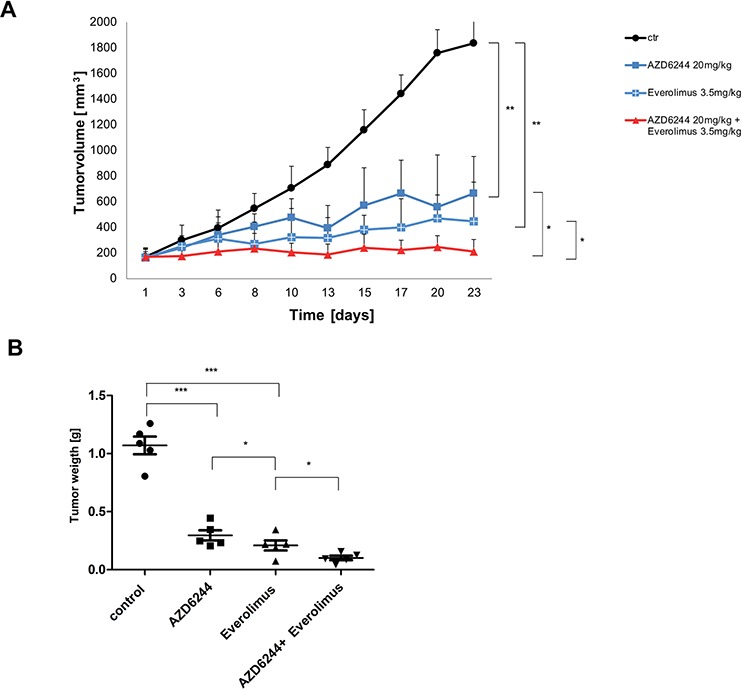
AZD6244 and Everolimus block tumor growth in-vivo as single agents and in combination **A.** HRAS mutant lung cancer cell line KNS-62 xenografts were treated with AZD6244 (20 mg/kg), or Everolimus 3.5 mg/kg), or a combination of AZD6244 and Everolimus (20 mg/kg and 3.5mg/kg, respectively), or vehicle (DMSO). Each group consisted of 5 mice and tumor volumes (mm^3^) are shown. Error bars represent standard error values and significance is (* = *p* < 0.05; ** = *p* < 0.01). **B.** Xenografts were explanted at the end of experiments and tumor weight was measured. Significance is (* = *p* < 0.05; ** = *p* < 0.01; *** = *p* < 0.001).

Next, we wanted to evaluate efficacy of MEK and mTOR inhibitors in a murine xenograft model. We tested several cell lines and found the human lung cancer cell line KNS-62 most suitable for xenotransplantation. Treatment of animals with oral AZD6244 or Everolimus resulted in a significant reduction in tumor growth *in-vivo* (Figure 6A). In addition, combination of AZD6244 and Everolimus even further reduced tumor growth *in-vivo* (Figure 6A). Of note, combination treatment was significantly more effective than single treatment for both agents (Figure 6A). These results were supported by determination of tumor weight at the end of the xenotransplantation. Again, tumor weight was significantly reduced in AZD6244 and Everolimus treated animals (Figure 6B) and tumor weight was significantly lower in the combination treatment arm (Figure 6B). We conclude that single and combination treatment of AZD6244 and Everolimus block tumor growth *in-vivo*.

## DISCUSSION

Lung adenocarcinomas have mutually exclusive mutations in receptor tyrosine kinase (RTK) and RAS pathway oncogenes such as EGFR and KRAS [1]. Further oncogenic mutations can be found in HRAS, NRAS, BRAF, ERBB2, MET, MAP2K1 and RIT1 [1, 27]. Targeting ‘driver’ mutations including EGFR in lung cancer and BRAF in melanoma resulted in great clinical success [28, 29]. Whereas targeting mutant KRAS remained difficult, several studies demonstrated that NRAS mutations can be targeted by inhibition of MEK in tumor cell lines [12, 13, 15]. Inhibition of MEK in NRAS mutant melanoma was also successful in a phase II trial and a large phase III trial is planned [16, 17]. In this study we were able to show for the first time that HRAS mutant cancer cell lines can be targeted by MEK kinase inhibition (Figure 1). Interestingly, the effect of susceptibility towards MEK inhibition could be observed for different background of cancer types. HRAS mutation conferred MEK sensitivity in lung cancer of adenocarcinoma and squamous origin, bladder cancer, endometrium cancer and squamous esophageal cancer cell lines (Figure 1). This is in accordance with mutations of NRAS that sensitize towards MEK inhibitors in different kind of tissues including lung cancer, neuroblastoma, melanoma and T-cell lymphoma [12, 13, 15]. HRAS and NRAS seem to share signaling similarities which cause also similar sensitivity towards targeted treatment. Both, HRAS and NRAS mutations activate the RAS and the mTOR pathway and induce sensitivity towards MEK inhibitors. All HRAS mutations studied here are at typical known sites for activating mutations including G12V and Q61L. We found that HRAS mutations at position G12 or Q61 rendered transformed Ba/F3 equally sensitive to MEK inhibitors indicating that all activing mutations at position G12 and Q61 might be eligible for targeted treatment patients with HRAS mutant tumors (Figure 1, 4). Other mutations outside this hotspots at position 12, 13 and 61 do occur but are rare (Figure 1, (COSMIC, 2015)). It is not clear whether HRAS mutations outside these hotspots are non-functional passenger mutations or rare activating mutations which have been identified e.g. for N- and KRAS in leukemia [30]. HRAS mutations are frequent with about 2.8 – 5.1% in lung and bladder cancer, however, rather infrequent in endometrium and esophageal cancer with about 1% each [31]. Yet our data indicates that, though rare, patients with HRAS mutated tumors of different tumor histology might benefit from targeted therapy. This was further promoted by experiments in which MEK inhibitors do not only cause growth inhibition but also induced apoptosis in HRAS mutant cancer cells (Figure 2). Targeting oncogenic HRAS by specific kinase inhibitors might be also relevant for tumors associated with so-called RASophathies. HRAS as proto-oncogene was associated with Costollo syndrome – a congenital disorder characterized by coarse face, loose skin, cardiomyopathy and predisposition to tumors such as benign papillomas or malign rhabdomyosarcomas, neuroblastoma and bladder cancer [32]. Patients, but not parents, showed mutations of HRAS at position glycine 12 and 13 [32]. However, targeting HRAS might be limited by development of resistance as seen for BRAF inhibitors in melanoma [33, 34].

To overcome possible resistance mechanisms and to expand the list of possible drugs we investigated combinations of PI3K or mTOR inhibitors with MEK inhibitors. For NRAS mutant cancers the combination of MEK and the dual PI3K/mTOR were described to be synergistic [14, 35, 36]. However, we found that inhibition of PI3K with the inhibitor BKM120 alone or in combination did not block cell growth or further enhanced it (Figure 3). The PI3K inhibitor BYL719 did not work synergistically either (data not shown). Our observations are supported by recent clinical data. The combination of BKM120 with MEK inhibitor Trametinib failed to achieve a reasonable response in RAS- or BRAF-mutant lung cancer patients in a phase Ib trial [37]. We speculated if mutant HRAS might signal to the mTOR complex directly without involving PI3K/AKT. This is reflected by our observations showing that the mTOR inhibitor Everolimus alone was sufficient to block cell growth in HRAS mutant cells compared to wild-type cell lines (Figure 4, 5). However, HRAS mutations seem to be a better predictive marker for MEK sensitivity compared to mTOR sensitivity. The effect of mTOR inhibitors on HRAS mutant cells was altogether less pronounced compared to MEK inhibitors. The effect of mTOR inhibitors was also minor compared to MEK inhibitors in NRAS mutant melanoma cells [14]. Yet, the combination of both drugs was synergistic and this synergism occurred at very low nanomolar concentrations which are also relevant for clinical treatment and might be associated with less toxicity (Figure 4, 5). Since most kinase inhibitors are in early clinical development, only some studies investigated combination thereof. One recent report demonstrated that the combination of AZD6244 and the AKT inhibitor MK2206 is tolerable in a phase I trial [38].

HRAS mutation status was barely tested within clinical trials. Early clinical trials investigating Everolimus in non-small cell lung cancer (NSCLC) showed partial responses (PR) for some patients, however, mutation status was not determined [39, 40]. A very recent trial testing AZD6244 in a KRAS/NRAS/HRAS mutant cohort reported one PR out of 10 patients included; however, the mutation status of these 10 specific patients treated with AZD6244 was not indicated [41].

We have shown that mutant HRAS is a potential drug target in different types of cancer including lung cancer and bladder cancer. HRAS mutations sensitize to single treatment with MEK and mTOR inhibitors which can be further enhanced by combinations of MEK and mTOR inhibitors. Thus, our data may encourage clinical studies on HRAS mutant cancer patients.

## MATERIALS AND METHODS

### Chemicals

Murine IL-3 was purchased from Sigma-Aldrich. AZD6244, MEK162 and PD0325901 were purchased from Selleck Chemical. Everolimus and AZD8055 were purchased from MedChemExpress. All inhibitors were solubilized in dimethyl sulfoxide (DMSO) at stock concentrations of 1 mM.

### Cell culture

T24 and KNS62 were purchased from National Institute of Biomedical lnnovation JCRB Cell Bank, Japan. NCI-H1915, Phoenix and RL952 were purchased from ATCC. KYSE-30, CAL-12T, HCC44, HCC78 and Ba/F3 were purchased from DSMZ (Deutsche Sammlung von Mikroorganismen und Zellkulturen), Germany. Lung cancer cell line NCI-H1915 cells were cultured in RPMI medium supplemented with 10% fetal calf serum (FCS) and 1 mM L-glutamine. KNS62, T24 and CAL12T cells were cultured in DMEM medium supplemented with 10% fetal calf serum and 1 mM L-glutamine. RL952 cells were cultured in DMEM and F12 medium (1:1) supplemented with 10% fetal calf serum, 0.005 mg/ml insulin (1:100 Opi) and 1 mM L-glutamine. KYSE30 cells were cultured in RPMI 1640 and F12 medium (1:1) supplemented with 10% fetal calf serum and 1 mM L-glutamine. Lung cancer cell lines HCC44 and HCC78 cells were cultured in RPMI medium supplemented with 10% fetal calf serum and 1 mM L-glutamine. Lung cancer cell line HCC827 cells were cultured in RPMI medium supplemented with 20% fetal calf serum and 1 mM L-glutamine. Ba/F3 cells were maintained in RPMI-1640 supplemented with 10% FCS and 0.5 ng/ml murine IL-3 (Sigma-Aldrich). For serum reduced experiments cell lines were kept in Accell serum free media (Dharmacon) supplemented with 1% FCS. For hypoxia studies cells were kept for 96 h in a 0.2% hypoxia chamber Invivo2 400 (Ruskinn).

### siRNA transfection and knockdown

KNS-62, NCI-H1915, T24, RL95–2, KYSE-30, CAL12T, HCC44, HCC827, and HCC78 were transfected with either control or two different siRNAs against HRAS. Accell siRNA was purchased from Dharmacon and used according to the manufacturer's protocol. In brief, siRNAs were diluted in Accell siRNA Delivery Media from Dharmacon to a final concentration of 1uM. Knock-down efficiency was observed after 96 h.

### Ba/F3 assays

Ba/F3 cells were transduced retrovirally with empty pMSCV-Puro (kindly provided by Dr. Balabanov) or pMSCV-Puro expression wild-type HRAS, HRAS Q61L, HRAS Q61R, HRAS G12V with the help of 293T Phoenix cells (ATCC). Synthesis of HRAS or HRAS mutants, cloning and DNA sequencing of cloned inserts was performed by LifeTechnologies. Transduced cells were seeded at concentrations below 1 × 10^6^/ml, selected with puromycin at 1 μg/ml (in addition with 0.1 ng/ml IL-3) 3d after transduction. Section pressure was maintained for at least 1 week, then IL-3 was reduced under ongoing puromycin treatment. IL-3 independent clones grew out after 5–7 days and were kept under puromycin 1 μg/ml.

### Western blot analysis

A total of 0.5 × 10^6^ cells were lysed for 30 minutes in ice-cold MPERM buffer supplemented with 25 mM NaF, 1 mM dithiothreitol, and complete protease inhibitor cocktail from Roche Diagnostics. Cell debris was removed by centrifugation at 10000 rpm for 10 minutes, loading buffer added and treated for 5 min at 95°C. Then, proteins were separated by gel electrophoresis. Separated proteins were blotted onto a nitrocellulose membrane (GE Healthcare) which was blocked with 5% bovine serum albumin in phosphate-buffered saline/Tween (0.05% Tween-20 in phosphate-buffered saline). The following antibodies were used: anti-phospho-ERK (*P*-p44/p42 (Tyr202/204, #9101, Cell Signaling Technology), anti-ERK (p44/p42, # 4695, Cell Signaling Technology), anti-phospho Akt (Ser473, # 4085, Cell Signaling Technology), anti-panAKT (# 9272, Cell Signaling Technology), anti-phospho MEK (Ser298, #9128, Cell Signaling Technology), anti-MEK (# 8727, Cell Signaling Technology), anti-phospho mTOR (Ser2448, #2971, Cell Signaling Technology),anti-mTOR (# 2972, Cell Signaling Technology), anti-HRAS rabbit (# ab97488, Abcam) and anti–actin (Sigma-Aldrich).

### PCR and sequencing of cell lines

Isolation of cellular RNA was done by using the Qiagen RNA Purification Kit (Qiagen). RNA (1 μg) was reverse transcribed into cDNA with a reverse transcription-PCR kit (Applied Biosystems). Next, 5 μL of cDNA was taken for a PCR of 50 μL volume. We used the following primers: HRAS forward, 5_-ggggcaggagaccctgtag-3_; HRAS reverse. PCR was performed, and 30 μL of PCR product was sent for sequencing to Microsynth, Switzerland. For sequencing, we used the same primers as for the PCR. Mutations can be found in Cancer Cell Line Encyclopedia (Encyclopedia, 2015).

### Apoptosis assays

Cell lines were treated with indicated concentrations of inhibitors and apoptosis was measured after 48 h and 72 h. Apoptosis was assessed by AnnexinV–APC (Enzo Lifescience) and propidium iodide (PI) (Sigma-Aldrich) by FACS. Both reagents were diluted to a final concentration of 1.5%. Specific cell death was calculated by the following equation: specific cell death % = (% experimental cell death - % spontaneous cell death)/(100% - % spontaneous cell death) x 100 (Jin et al., 2006).

### Cell proliferation and viability assays

Cell proliferation was determined by Cell-Titer-Glo Reagent (Promega) according to the manufacturer's instructions. Cells were plated in 96-well plates at a density of 500 - 2500 cells per well. The next day, drugs were added at indicated concentrations and cell proliferation was measured 4 days later. Measurement of proliferation was done using a 96-well plate luminometer/plate reader (Synergy 2, Biotek). Data were calculated as relative values: luminescence for a given drug concentration was compared to luminescence of untreated cells. All experimental points were set up in duplicate replicates and were conducted at least 3 independent times. IC50 were calculated with GraphPad Prims. Combination index (CI) values with CalcuSyn Software (Biosoft) according to Chou-Talalay [18]. CI values less than one were considered synergistic.

### Mouse xenotransplantation

We tested lung cancer cell line KNS-62 and bladder cancer cell line T24 for engraftment rates by subcutaneous injection in CB17 SCID−/− mice. Engraftment and growth rate was excellent for KNS-62 already after 6 days but poor for T24 cells (tumors shrinked after 14d). Finally, we xenotransplanted 1*10^6^ KNS-62 cells into CB17 SCID−/− mice. Engrafted tumors reached 150 mm^3^ after 6 days and animals were randomized into 4 groups: DMSO only (*n* = 5), AZD6244 (Selumetinib) at 20mg/kg (*n* = 5), Everolimus at 3.5mg/kg (*n* = 5), and combined AZD6244 and Everolimus at 20mg/kg and 3.5mg/kg respectively (*n* = 5). Inhibitors were kept at stock concentrations of 250 mM (AZD6244) and 10 mM (Everolimus), diluted in 1000ul DMSO each and carefully diluted in animal water. Concentrations were adjusted to 4ml drink volume per day which was the observed volume for previous experiments. Tumor size and weight was monitored three times weekly, and the mouse technician who was measuring tumor size was blinded to grouping until the trial was completed. Determination of tumor volume was done by 3 dimensional digital caliper measurements. Statistical analysis of the experiment was done by a two-tailed t test at each time point. *P*-values < 0.05 (*), or *p*-values < 0.01 (**) were considered significant between different treatment groups. The animal study was performed in accordance with the University Zurich Animal Committee and animals were euthanized as soon as tumor volume exceeded 3000 mm^3^ or necrosis of tumors.

## SUPPLEMENTARY FIGURES


