# Supplementary material for: Mutant HRAS as novel target for MEK and mTOR inhibitors
Source: Oncotarget. 2015 Nov 3;6(39):42183–96. doi: 10.18632/oncotarget.5619 (PMC4747218; doi:10.18632/oncotarget.5619)
Supplement: Supplementary file 1 [file oncotarget-06-42183-s001.pdf]

SUPPLEMENTARY FIGURES

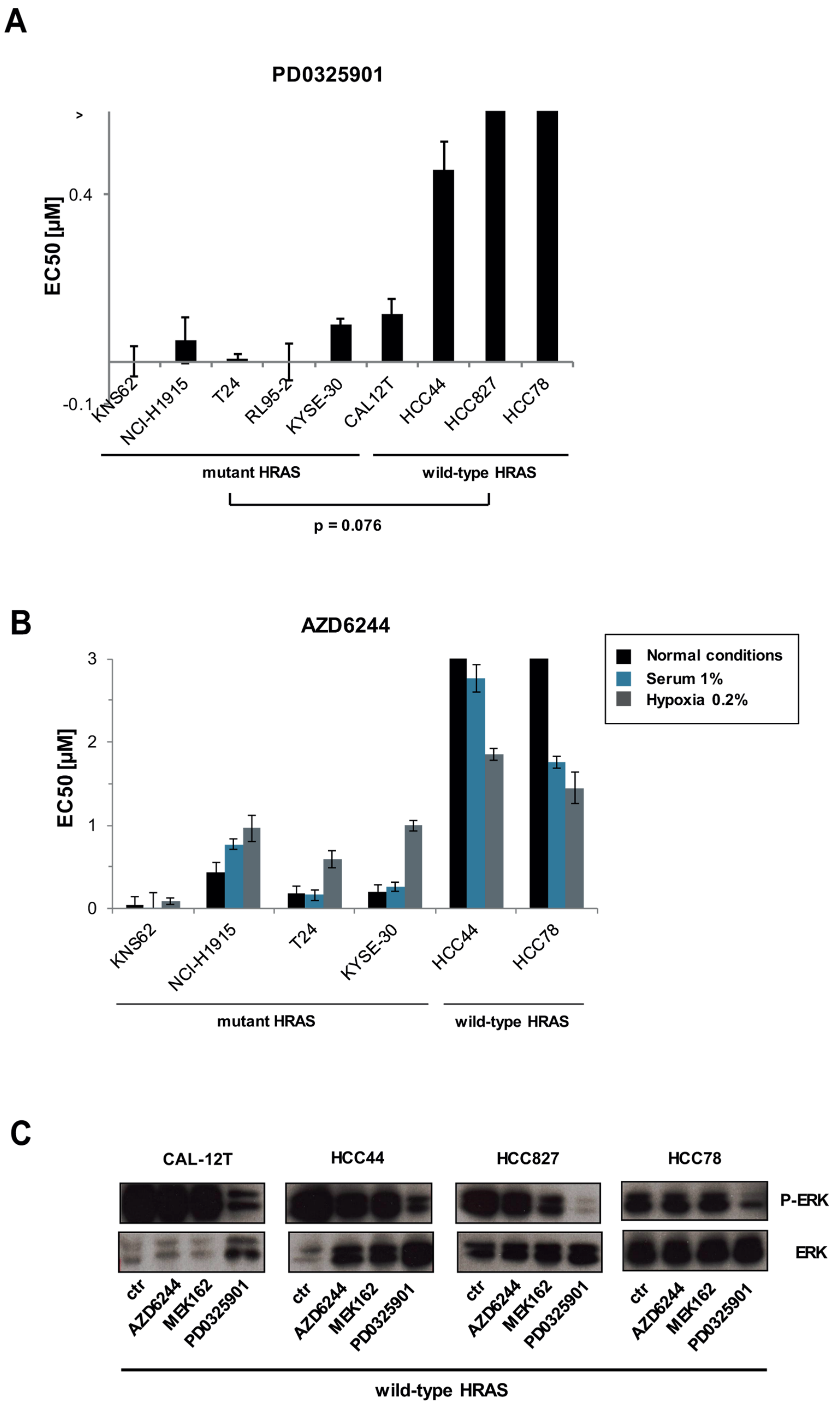

Supplementary Figure S1: MEK inhibition by PD0325901 blocks cell growth in HRAS mutant cells. A. Mutant HRAS und wild-type HRAS cell lines were treated with 6 increasing concentrations of PD0325901 for 96 hours. Then, cell growth was measured by Cell Titer Glo according to the manufacturer's instructions. EC50 values were calculated with GraphPad Prism and depicted at bars. Statistical significance between mutant and wild-type cell lines was calculated with student's *t*-test. **B.** Mutant HRAS und wild-type HRAS cell lines were treated with 6 increasing concentrations of AZD6244 for 96 hours. However, cells in "serum 1%" were resuspended in serumfree media (Dharmacon) supplemented with 1% FCS and cells under "hypoxia 0.2%" were transferred to a hypoxia chamber for 96 h. Then, results were measured in parallel after 96 h by Cell Titer Glo according to the manufacturer's instructions. EC50 values were calculated with GraphPad Prism and depicted at bars. **C.** All cell lines were kept under equal conditions, then treated with 500nM of AZD6244, MEK162 and PD0325901 for 1 hour and next lysed and subjected to Western blot. Phosphorylation levels of ERK and MEK were detected by specific anti-phospho antibodies. Loading was verified by specific antibodies to total ERK, MEK and anti–actin.

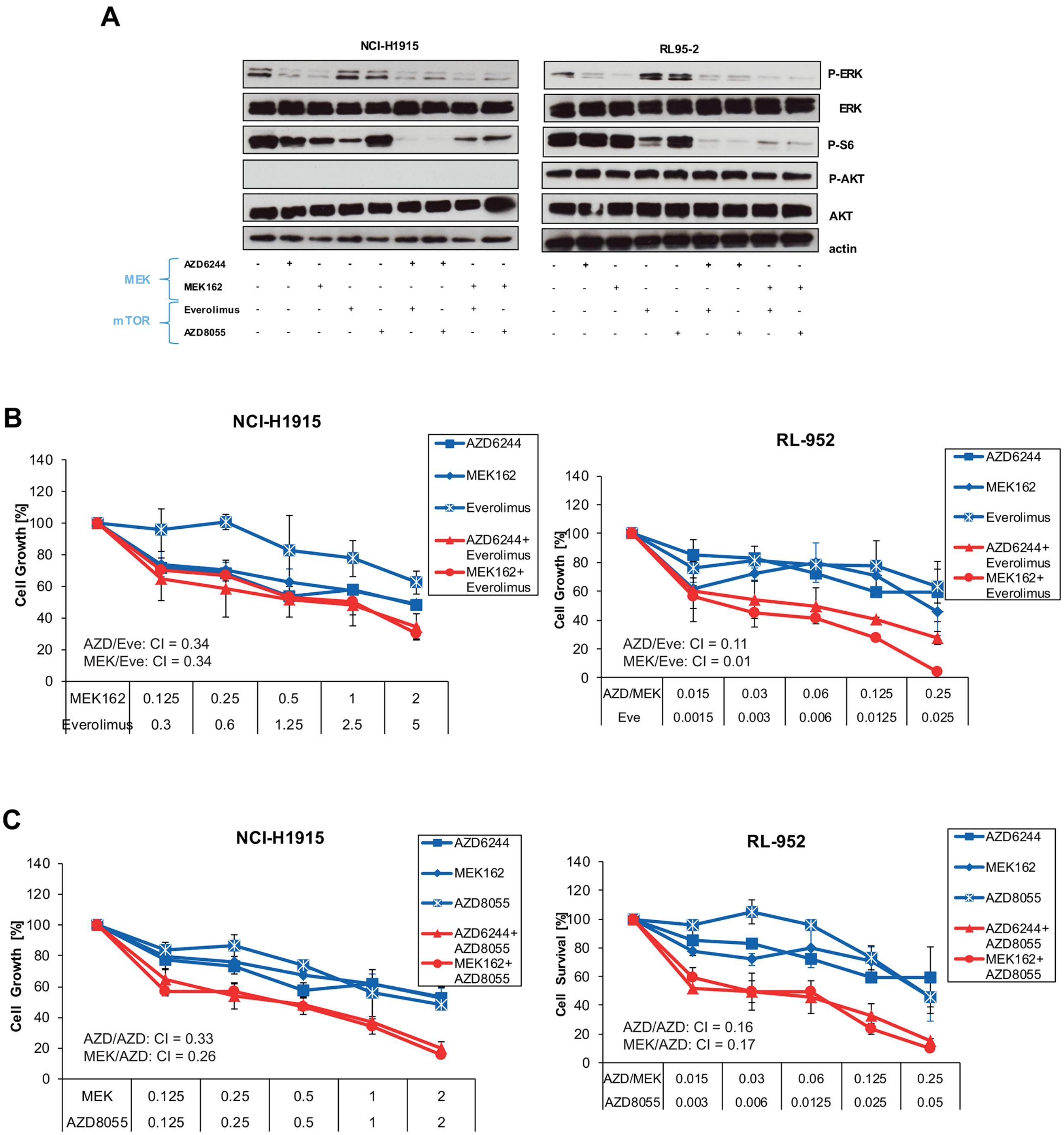

Supplementary Figure S2: Inhibition of the mTOR pathway blocks cell growth in HRAS mutant cell lines NCI-H195 and RL95–2. A. NCI-H1915 and RL95–2 cells were treated with 250nM AZD6244, 250nM MEK162, 5nM of Everolimus, 250nM AKT8055 or combinations thereof as indicated for 1 hour. Then, cells were lysed and analysed by Western blot. **B.** NCI-H1915 and RL95–2 cells were left untreated or treated with indicated concentrations of Everolimus and AZD6244/MEK162 for 96 hours. Then, cell growth was measured by Cell Titer Glo according to the manufacturer's instructions. CIs are indicated. **C.** Same as in B., but the mTOR inhibitor AZD8055 was used instead of Everolimus.

A

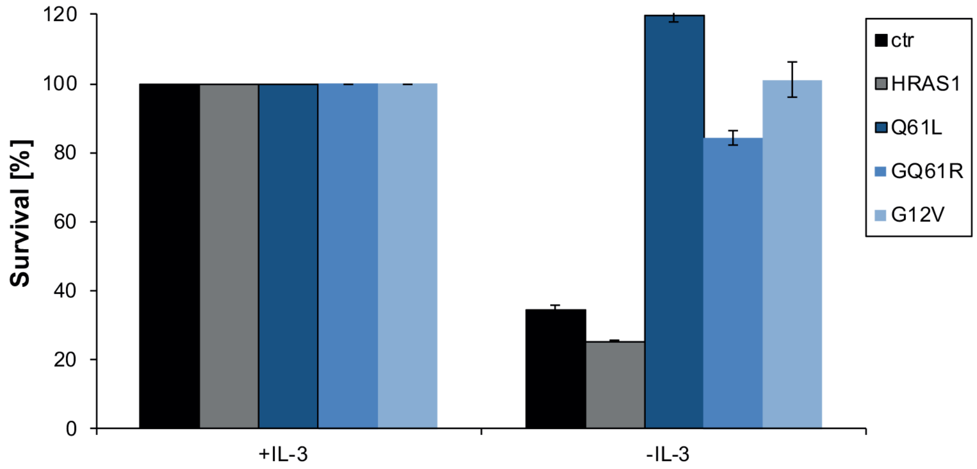

Supplementary Figure S3: Only mutant HRAS can transform Ba/F3 cells. A. Ba/F3 cells or Ba/F3 cells transduced with empty vector or HRAS Q61L, Q61R or G12V mutations were seeded in IL-3 reduced media with 2 pg/ml IL-3. 96 h later cell growth was measured by Cell Titer Glo.
